# Assembly of forest communities across East Asia – insights from phylogenetic community structure and species pool scaling

**DOI:** 10.1038/srep09337

**Published:** 2015-03-23

**Authors:** Gang Feng, Xiangcheng Mi, Wolf L. Eiserhardt, Guangze Jin, Weiguo Sang, Zhijun Lu, Xihua Wang, Xiankun Li, Buhang Li, Ifang Sun, Keping Ma, Jens-Christian Svenning

**Affiliations:** 1 Section for Ecoinformatics and Biodiversity, Department of Bioscience, Aarhus University, Ny Munkegade 114, DK-8000 Aarhus CDenmark; 2 State Key Laboratory of Vegetation and Environmental Change, Institute of Botany, Chinese Academy of Sciences, Beijing; 3 Royal Botanic Gardens, Kew, TW9 3AB Richmond, Surrey, UK; 4 Center for Ecological Research, Northeast Forestry University, Harbin; 5 Key Laboratory of Aquatic Botany and Watershed Ecology, Wuhan Botanical Garden, Chinese Academy of Sciences, Wuhan; 6 Tiantong National Field Observation Station for Forest Ecosystem, East China Normal University, Shanghai; 7 Guangxi Institute of Botany, Chinese Academy of Sciences, Guilin; 8 State Key Lab of Biological Control and School of Life Sciences, Guangdong Key Lab of Plant Resources, SYSU-Alberta Joint Lab for Biodiversity Conservation, Sun Yat-sen University, Guangzhou; 9 Department of Natural Resources and Environmental Studies, National Dong Hwa University, Hualein

## Abstract

Local communities are assembled from larger-scale species pools via dispersal, environmental filtering, biotic interactions, and local stochastic demographic processes. The relative importance, scaling and interplay of these assembly processes can be elucidated by comparing local communities to variously circumscribed species pools. Here we present the first study applying this approach to forest tree communities across East Asia, focusing on community phylogenetic structure and using data from a global network of tropical, subtropical and temperate forest plots. We found that Net Relatedness Index (NRI) and Nearest Taxon Index (NTI) values were generally lower with geographically broad species pools (global and Asian species pools) than with an East Asian species pool, except that global species pool produced higher NTI than the East Asian species pool. The lower NRI for the global relative to the East Asian species pool may indicate an important role of intercontinental migration during the Neogene and Quaternary and climatic conservatism in shaping the deeper phylogenetic structure of tree communities in East Asia. In contrast, higher NTI for the global relative to the East Asian species pool is consistent with recent localized diversification determining the shallow phylogenetic structure.

The recent integration of phylogeny and community ecology is bridging the gap between biogeography and ecology, providing novel insights for ecological and biogeographic theory, notably as regards the relative roles of local and regional factors in community assembly^1^,^2^,^3^,^4^. Importantly, the tropical niche conservatism hypothesis, which proposes that communities in low-latitude warm areas are dominated by older clades than communities in high-latitude cold regions, has been developed and supported by studies linking phylogenies to local communities^5^,^6^,^7^,^8^. The tropical niche conservatism hypothesis suggests that most lineages originated in the warm tropics and have been limited in their ability to disperse into the colder areas because of temperature niche conservatism^5^.

When linking phylogenies to local communities for describing community phylogenetic structure^9^, geographic scope is crucial^10^. Notably, estimates of community phylogenetic structure involve comparing realized community to null-model communities^1^. A geographic scope is needed for defining the null model, i.e., how far beyond the local community does one go in the null-model sampling, and varying it may provide insights on community assembly^10^. Importantly, the choice of geographic scope for the species pool may shed light on the scale of the diversification and migration processes by which the communities have been built^9^,^10^. Increasing the geographic scope of the species pool should produce more clustered phylogenetic community structure if localized diversification and limited migration has dominated. In contrast, if assembly has involved intercontinental migration and climatic conservatism, increasing the geographic scope of the species pool may not lead to increasing phylogenetic clustering or could even produce decreasing clustering, if close relatives tend to not co-occur locally, but instead show disjunct distributions^10^,^11^. We note that these patterns of relatedness, where related taxa have disjunct occurrences often occur at genus level or higher taxonomic levels, reflecting migrations occurred millions of years ago^12^. Climatic conservatism is necessary component in maintaining such disjunctions, as the otherwise would be able to survive or recolonize the intervening areas.

Species' abundance in local communities is an important component of community structure and is suggested to be primarily controlled by contemporary local factors such as competition, abiotic stress, and disturbances^13^,^14^,^15^. Comparing indices of community structure with or without abundance weighting may thereby provide insights into community assembly^16^ and should also better relate to ecosystem functioning^17^,^18^.

East Asia is naturally largely forest-covered and includes a continuum of tropical, subtropical, temperate and boreal forests^19^,^20^, making it an ideal place to study assembly of tree communities across climate gradients. Moreover, many temperate and subtropical genera or pairs of closely related genera as well as even a few species show disjunctions between East Asia and eastern North America (sometimes referred to as ‘Asa Gray disjunctions', after the 19^th^ century botanist who first reported them), reflecting intercontinental migration and subsequent diversification as an important component in the build-up of these floras^12^,^21^. The disjunctions to a large extent reflect pre-Quaternary Cenozoic high-latitude migrations of temperate and subtropical taxa, subsequently contracting southwards due to Neogene and Quaternary cooling^12^,^21^. Further, topographically-driven localized diversification within East Asia may be a key driver of its higher diversity relative to eastern North America^22^, although less Neogene and Quaternary extinction also plays a role^23^. So far no studies have quantitatively assessed the relative roles of processes at contrasting scales in determining community phylogenetic structure in East Asian forests.

In this study, linking phylogenetic community structure of 20 forest plots, ranging from tropical to temperate areas in Mainland China and Taiwan, to minimum temperature in the coldest month, we first tested if community phylogenetic clustering increased with increasing coldness, as predicted under tropical niche conservatism^5^. To assess the relative roles of intercontinental migration, climatic conservatism and local diversification in these forest-tree community assembly, we then compared the effect on community phylogenetic structure of three differentially circumscribed species pools (East Asian, Asian, and global). To assess the potential roles of local factors in community assembly, we furthermore assessed the effect of species abundance on community phylogenetic structure.

## Results

**Figure 1:**
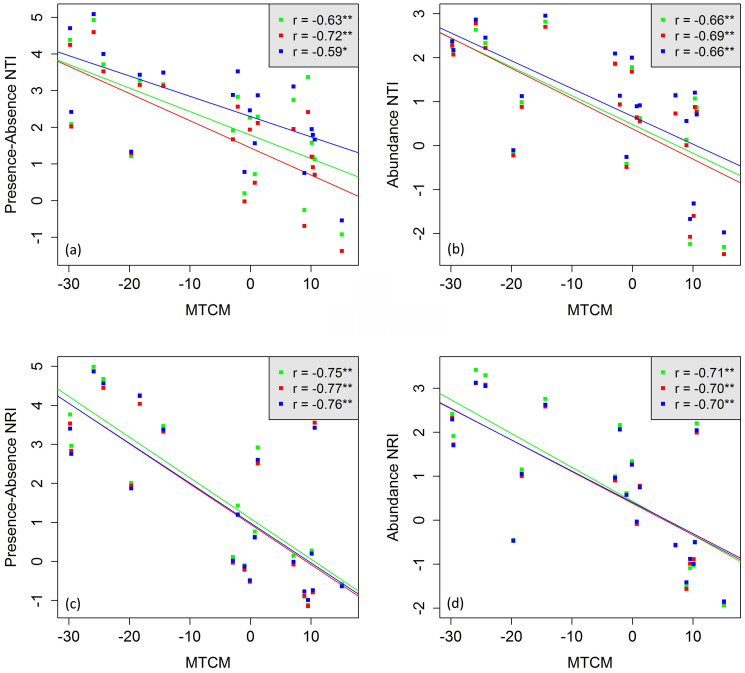
Scatter plots of NTI/NRI against minimum temperature of coldest month (MTCM). Species pool: green = East Asian, red = Asian, and blue = global. (a) is the presence-absence based NTI; (b) is the abundance based NTI; (c) is the presence-absence based NRI; (d) is the abundance based NRI. Spearman correlations and linear regression fits are given (* p < 0.05, ** p < 0.01).

Generally, for all species pools and for both presence/absence data and abundance data, we found a significantly negative correlation between NRI/NRI and minimum temperature of the coldest month (Fig. 1), i.e., increasing phylogenetic clustering with increasingly cold winters.

**Figure 2:**
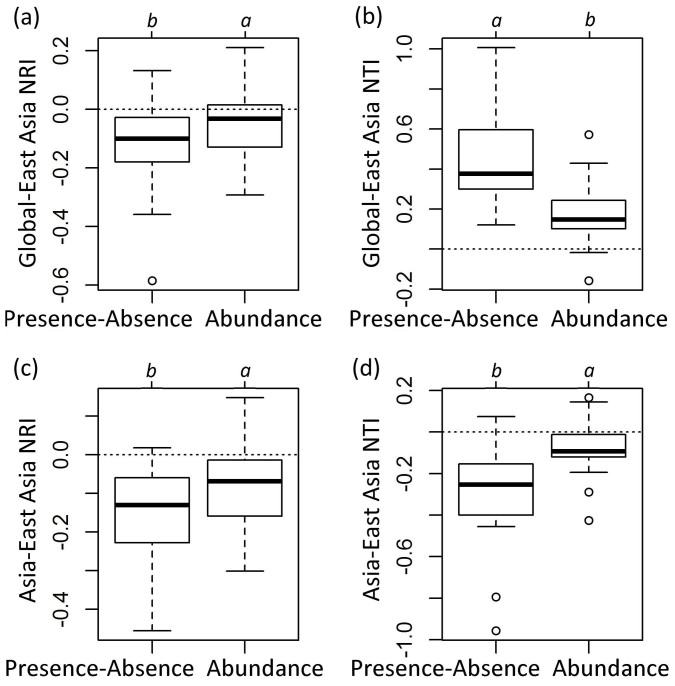
Differences in NTI/NRI among species pools. Global-East Asia means values calculated with the global species pool minus values calculated with the East Asian species pool; Asia-East Asia means values calculated with the Asian species pool minus values calculated with the East Asian species pool. (a) is Global-East Asia NRI; (b) is Global-East Asia NTI; (c) is Asia-East Asia NRI; (d) is Asia-East Asia NTI. Different letters indicate significant differences in NRI/NTI between abundance-weighted and presence/absence based indices (P < 0.05).

Differences between NRI for global vs. East Asian and Asian vs. East Asian species pools, and NTI for global vs. East Asian species pools were significantly smaller than zero, i.e., phylogenetic structure was more over-dispersed with larger species pool. In contrast, the differences between NTI for global vs. East Asian species pools were significantly larger than zero, i.e., this single aspect of community phylogenetic structure became more clustered with increasing geographic scope of the species pool (Fig. 2).

Compared with presence/absence-based community phylogenetic structure, abundance-weighted produced significantly smaller differences when varying the geographic scope of the species pools (Fig. 2).

## Discussion

To our knowledge, this is the first study to link phylogenetic community ecology with the species pool hypothesis to evaluate the geographic variation of phylogenetic structure in forest-tree communities across East Asia. This region is a global biodiversity hotspot, and its forests range from tropical to boreal regions. Across these forests, we found evidence for a strong role of tropical niche conservatism in community assembly. We furthermore found that broad-scale deep-time intercontinental migration jointly with climatic niche conservatism appear to have left a strong signature in East Asian forests' tree community phylogenetic structure, with also a shallow phylogenetic imprint of local diversification. Finally, accounting for local abundances reduced the effects of species pools on community structure, suggesting a strong role of local factors in shaping local dominance patterns.

Several studies have found a more clustered phylogenetic structure in temperate regions than tropical areas, covering a wide range of organisms, e.g., butterflies, ants, tree frogs and trees^6^,^7^,^24^,^25^,^26^. However, most of these studies were conducted in North America and so far no studies have tested this in East Asia. This study, covering tropical, subtropical, temperate and boreal forest in this region, found a significantly increasing phylogenetic clustering with decreasing minimum temperature in the coldest month, providing strong support for the role of tropical niche conservatism in forest-tree community assembly across this biodiversity hotspot region.

A strong floristic affinity between tropical rain forests in China and the rest of Asia has been widely documented and is suggested to be driven by the continuous land connections between China and tropical Southeast Asia throughout the Cenozoic^27^,^28^,^29^. The increasing phylogenetic over-dispersion (represented by both Net Relatedness Index (NRI) and Nearest Taxon Index (NTI)) when enlarging the geographic scope of the species pool from East Asia to include both East and Southeast Asia may indicate the role of continuous inter-regional migration throughout the Cenozoic in shaping Chinese forest tree-community structure.

These widely recognized disjunct distributions of plants between East Asia and eastern North America suggest that floras in these regions (and across the Northern Hemisphere) were formerly highly connected and built up via intercontinental migrations and subsequent diversification^12^,^21^,^22^. Furthermore, previous phylogenetic analyses have shown that further local diversification of species has occurred in both East Asia and eastern North America after the formation of the disjunction, with a few disjuncts even diversified into different genera^12^. Pantropical intercontinental disjunctions of plants, for instance, from Africa to Asia, from South America to Asia through North America, have also been widely reported^30^,^31^,^32^. Hence, these studies together suggest a scenario dominated by intercontinental migration, climatic niche conservatism (hindering survival or recolonization of the intervening areas), notably with respect to the deeper-level community phylogenetic structure, and subsequent local diversification, mostly within genera^12^. Such a scenario is indeed consistent with our species pool scaling results, where we found decreasing phylogenetic clustering or increasing over-dispersion (represented by NRI) with increased geographic scope of the species pool, from East Asia to global. In contrast, the significantly more positive NTI with increasing geographic species pool scope (from East Asia to global) is consistent with a role for recent local diversification in determining community structure in East Asian forests, as NTI reflects shallow (recent) phylogenetic structure.

Previous studies at local scales have reported evidence for the roles of competitive exclusion, small-scale habitat filtering and local dispersal limitation in shaping community phylogenetic structure in East Asian tropical to temperate forests^33^,^34^,^35^. In the present study, the smaller differences in phylogenetic structure among species pools when species abundances were considered may indicate a strong role of such local factors in controlling local dominance patterns and apparently with little coupling to long-term lineage diversification and migration history. Analogously, a phylogenetic study of microbial communities compared patterns produced by the qualitative and quantitative indices, and concluded that the quantitative indices are suitable to reflect the changes caused by transient environmental changes, while the qualitative indices are suitable to indicate the factors limiting the general survival of bacteria^15^. Previous phylogenetic studies on plant communities also found that abundance-weighted indices reflected different patterns and better relate to ecosystem functions, i.e., vegetation productivity than qualitative indices^17^,^18^.

Given the global nature of this study and the more than 6,000 species covered, we had to use a fairly coarse-grained phylogeny. This should not bias our results, but means that it is the mostly the deeper phylogenetic relations that will have been able to influence the phylogenetic assemblage patterns. This should matter little for especially the NRI results, as this index in any case mostly reflects the deeper part of a phylogeny^36^. Notably, the famous Eastern Asian and eastern North American disjunct distributions primarily involve patterns at the genus level and above^12^. Furthermore, with decreasing phylogenetic resolution NRI and NTI will tend towards zero^36^, i.e., simply leading to less power for detecting significant community structure, which was no problem in this case.

Analyzing climatic correlates and species pool scaling effects on community phylogenetic structure provided novel insights into the processes that have led to the assembly of rich forest-tree communities in East Asia. Notably, our study shows that these communities show imprints of deep-time broad-scale diversification and migration processes, more recent localized diversification, as well as contemporary local ecological processes.

## Methods

### Community composition

**Figure 3:**
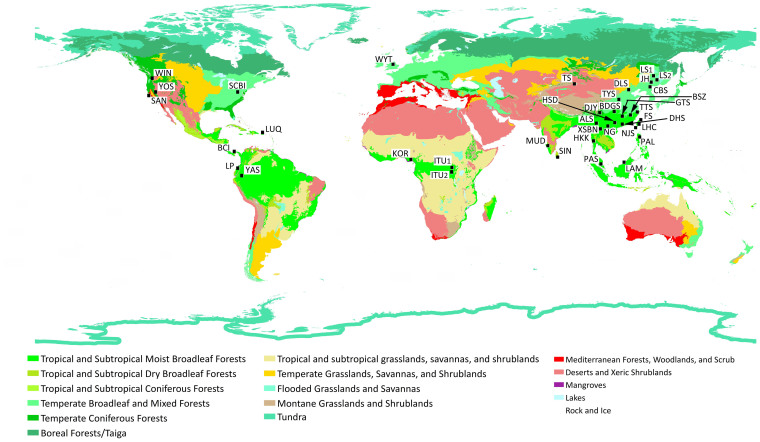
Distribution of the 38 forest plots. The 20 plots in Mainland China and Taiwan are Nanjenshan (NJS), Lienhuachih (LHC), Fushan (FS), Dinghushan (DHS), Heishiding (HSD), Nonggang (NG), Xishuangbanna (XSBN), Ailaoshan (ALS), Baishanzu (BSZ), Gutianshan (GTS), Tiantongshan (TTS), Badagongshan (BDGS), Dujiangyan (DJY), Taiyueshan (TYS), Tianshan (TS), Donglingshan (DLS), Changbaishan (CBS), Jiaohe (JH), Liangshui_1_ (LS_1_), Liangshui_2_ (LS_2_). The 18 plots outside of Mainland China and Taiwan are Palanan (PAL), Lambir (LAM), Pasoh (PAS), HuaiKhaKhaeng (HKK), Sinharaja (SIN), Mudumalai (MUD), Korup (KOR), Ituri_1_ (ITU_1_), Ituri_2_ (ITU_2_), Wytham Woods (WYT), Wind River (WIN), Yosemite (YOS), Santa Cruz (SAN), SCBI, Barro Colorado Island (BCI), Luquillo (LUQ), Yasuni (YAS), La Planada (LP). The background shows the distribution of 14 biomes on the world^46^. The map was plotted using ArcGIS 10.1 (http://www.esri.com/).

Species list and species abundance of 20 forest plots in Mainland China and Taiwan mainly came from the Chinese Forest Biodiversity Network (CForBio, http://www.cfbiodiv.org/index.asp) and Taiwan Forest Dynamics Plots Network, with several additional plots from other published research (Table A1, Fig 3). Data on the other 18 plots outside of this area came from Center for Tropical Forest Science (CTFS, http://www.ctfs.si.edu/, Table A2, Fig 3). All woody plant individuals with diameter at breast height ≥1 cm were recorded in each plot. The 20 plots in Mainland China and Taiwan covered 1,840 species with 1,556,462 individuals.

### Phylogeny

We assembled a phylogenetic tree including all angiosperm species that occurred in the 38 forest plots using Phylomatic^37^, based on the R20120829 Phylomatic tree. Gymnosperms were excluded because they would strongly bias metrics of community phylogenetic structure^38^,^39^. Phylogenetic relationships were based on the results of the Angiosperm Phylogeny Group III^40^. Branch length was calibrated in Phylocom 4.2^41^ using the bladj algorithm. The ages file used here was from Gastauer and Meira-Neto^42^ to avoid the inconsistences of syntax in the ages file provided by Phylocom 4.2.

### Climate

To test the relation between phylogenetic structure and climate variables, we used minimum temperature of the coldest month (MTCM) from the WorldClim database^43^, as this variable is closely related to the cold stress mechanism hypothesized in the tropical niche conservatism hypothesis^44^. Previous studies have found it to be the best variable for explaining geographic variation of community phylogenetic structure in North American forests^6^,^7^. MTCM values were extracted from the 30 arc-seconds resolution layer at the coordinates of each forest plot using ArcGIS 10.1.

### Species pools

We defined three nested species pools to be used as source pools in our null models of community assembly (see below). The smallest East Asian species pool (covering forest plots from Mainland China as well as Taiwan) included all 1,719 species (1,495,008 individuals) from 20 plots (Table A1). The Asian species pool included all 3,380 species from the 26 plots in South and East Asia, and the global species pool included all 6,091 species occurring in the total set of 38 plots.

### Analyses

The Net Relatedness Index (NRI) and the Nearest Taxon Index (NTI)^1^ were used to quantify phylogenetic structure. NRI measures the mean pairwise phylogenetic distance between all species or individuals in a sample (MPD), while NTI measures the mean phylogenetic distance between a species or individual and its closest relative MNTD, in both cases adjusting for the null-model expectation by random sampling from a species pool. They are calculated as
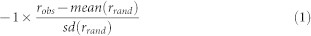
*r_obs_* is the observed NRI/NTI; *r_rand_* is the MPD/MNTD from a null model, which is built by permuting the species labels across a phylogeny covering all species in a given species pool and using the “taxa.labels” null model in picante, to preserve the community structure and have a reliable randomization^45^. Positive values mean phylogenetic clustering, where species in the community are more closed related than expected, while negative values mean phylogenetic over-dispersion, where species in the community are more distantly related than expected. NRI primarily reflects structure at deeper parts of phylogeny, while NTI mainly reflects shallow parts of phylogeny^1^,^36^. NRI and NTI were computed both using species presence/absence (unweighted) and abundance (abundance-weighted).

We calculated the NRI and NTI of each forest plot based on the three species pools, i.e., East Asian species pool, Asian species pool, and global species pool. Pearson correlations were used to test the relations between NRI/NTI and MTCM. We then computed the differences between the NRI/NTI calculated with different species pools, i.e., NRI/NTI produced by global species pool minus NRI/NTI produced by East Asian species pool, and NRI/NTI produced by Asian species pool minus NRI/NTI produced by East Asian species pool. We also calculated the differences between each pair of abundance-weighted and unweighted indices. Either Student's t test or Wilcoxon rank-sum test was used to test if these differences were significantly different from zero, depending on the error distribution (normal vs. non-normal, respectively).

## Author Contributions

G.F. and J.-C.S. designed the study, conducted the analyses and wrote the paper. X.C.M., G.Z.J., W.G.S., Z.J.L., X.H.W., X.K.L., B.H.L., I.F.S. and K.P.M. wrote the paper and contributed the data. W.L.E. discussed and wrote the paper.

## Supplementary Material

Supplementary Information Assembly of forest communities across East Asia - insights from phylogenetic community structure and species pool scaling
