# Supplementary material for: Assembly of forest communities across East Asia – insights from phylogenetic community structure and species pool scaling
Source: Sci Rep. 2015 Mar 23;5:9337. doi: 10.1038/srep09337 (PMC4369734; doi:10.1038/srep09337)
Supplement: Supplementary Information — Assembly of forest communities across East Asia - insights from phylogenetic community structure and species pool scaling [file srep09337-s1.pdf]

Assembly of forest communities across East Asia – insights from phylogenetic community structure and species pool scaling

Gang Feng[1*], Xiangcheng Mi[2], Wolf L. Eiserhardt[1], Guangze Jin[3], Weiguo Sang[2], Zhijun Lu[4], Xihua Wang[5], Xiankun Li[6], BuhangLi[7], Ifang Sun[8], Keping Ma[2], Jens-Christian Svenning[1]

Appendices

Table A1. General information on the 20 forest plots in Mainland China and Taiwan. The low numbers of stems (No. Stem) in TS and TYS are mainly driven by the strong dominance of gymnosperms. Minimum temperature in the coldest month (MTCM) ranges from -29.8 to 15.1. See Fig. 3 for abbreviations.

	Species Richness	No. Stems	Longitude	Latitude	MTCM
ALS	67	12,126	101.03	24.54	-0.1
BDGS	235	187,249	110.09	29.77	-2.9
BSZ	151	30,866	119.20	27.76	-2.1
CBS	49	36,427	128.08	42.38	-24.3
DHS	207	71,408	112.51	23.17	7.1
DJY	105	16,846	103.45	30.73	1.2
DLS	53	103,431	115.43	39.96	-18.3
FS	107	111,851	121.56	24.76	10.1
GTS	157	137,770	118.12	29.25	-1
HSD	232	264,846	111.53	23.27	8.9
JH	47	51,593	127.74	43.96	-25.9
LHC	142	150,760	120.88	23.91	10.3
LS_2	44	19,066	128.89	47.18	-29.6
LS_1	34	11,726	128.85	47.20	-29.8
NG	219	67,753	106.95	22.43	10.6
NJS	119	30,967	120.85	22.06	15.1
TS	8	103	87.47	43.43	-19.7
TTS	149	94,550	121.79	29.81	0.7
TYS	20	245	112.15	36.69	-14.4
XSBN	461	95,425	101.58	21.61	9.5

Table A2. Information on the 18 forest plots outside Mainland China and Taiwan. See Fig. 3 for abbreviations.

	Species Richness	Longitude	Latitude
LUQ	136	-65.82	18.32
BCI	302	-79.85	9.15
LP	153	-77.99	1.16
YAS	1,018	-76.4	-0.69
PAL	292	122.39	17.04
HKK	263	99.22	15.63
MUD	67	76.53	11.6
SIN	199	80.4	6.4
LAM	1,010	114.02	4.19
PAS	752	102.31	2.98
KOR	328	8.85	5.07
ITU$_1$	363	28.58	1.44
ITU$_2$	370	28.58	1.44
YOS	22	-119.82	37.77
WYT	15	-1.34	51.77
SCBI	59	-78.15	38.89
WIN	24	-121.96	45.82
SAN	26	-122.08	37.01
